# Sleep Quality and Depression and Their Association with Other Factors in Hemodialysis Patients

**DOI:** 10.5539/gjhs.v8n8p121

**Published:** 2015-12-17

**Authors:** Masomeh Norozi Firoz, Vida Shafipour, Hedayat Jafari, Seyed Hamzeh Hosseini, Jamshid Yazdani Charati

**Affiliations:** 1 Student of M.S Nursing, School of Nursing and Midwifery, Mazandaran University of Medical Science, Sari, Iran; 2 Department of Medical-Surgical Nursing, School of Nursing and Midwifery, Mazandaran University of Medical Sciences, Sari, Iran; 3 Psychiatry and Behavioral Sciences Research Center, Addiction Institute, Mazandaran University of Medical Sciences, Sari, Iran; 4 Department of Biostatistics, Faculty of Health, Mazandaran University of Medical Sciences, Sari, Iran

**Keywords:** depression, sleep quality, hemodialysis

## Abstract

**Background:**

Sleep disorders and depression, accompanied by reduced quality of life and increased mortality are the most common psychological problems in dialysis patients. This study was conducted with the aim to investigate depression and sleep quality and their association with some demographic and clinical factors in hemodialysis patients.

**Method:**

This descriptive-correlative study was conducted on 310 patients undergoing hemodialysis in 8 centers in educational hospitals in Mazandaran University of Medical Sciences. Data collection tools included a demographic questionnaire, Beck Depression Inventory, and Pittsburg Sleep Quality Index (PSQI). Statistical analysis was conducted using Chi-Square test and regression model.

**Results:**

Results obtained showed 44.8% depression in patients. Significant relationships were found between depression and increased blood phosphorus (P=0.002) and urea (P=0.001). Poor sleep quality was observed in 73.5% of hemodialysis patients, which was found significantly related to aging (P=0.048), female (P=0.04), and reduced frequency of weekly hemodialysis (P=0.035).

**Conclusion:**

Depression and poor sleep quality are two common factors in hemodialysis patients, but patients do not overtly show symptoms of these disorders.

## 1. Introduction

Hemodialysis is an alternative treatment of end-stage renal disease (ESRD)(Turkmen et al., 2012). According to the 2014 report of the Center for Research in Renal Diseases and Kidney Transplantation of Iran, of about 32686 patients with ESRD in Iran, 15957 receive hemodialysis (Retrieved May 4, 2015, from http://WWW.sportmedicine.ir/modulesiphp=contnts8t=37). Constant stress caused by not only the disease, but also the development of functional and nutritional limitations, medication side-effects, comorbid chronic diseases, changes in self-perception, and fear of death, predispose these patients to numerous emotional problems (Trbojević-Stanković et al., 2014) including depression (Son, Choi, Park, Bae, & Lee, 2009). While the exact prevalence of depression among hemodialysis patients is unclear, it has been estimated at 10%-66 (Spahbodi, Hosseini, Makhloogh, Sadeghie, & Taghipoor, 2014). Hedaiati et al. found depression to be four times more likely in hemodialysis patients than in the general population (Hedayati et al., 2008). The majority of hemodialysis patients suffer from poorer quality of life, more co-morbid conditions, more functional impairments, and higher mortality rates (Battistello, 2012; Lægreid, Aasarød, Bye, Leivestad, & Jordhøy, 2013). Despite its prevalence in hemodialysis patients, depression usually remains underdiagnosed due to its overlapping symptoms with both uremia and a number of physical conditions such as loss of appetite, fatigue, lethargy, loss of libido, and impaired sleep pattern (Sadock & Sadock, 2011; Trbojević-Stanković et al., 2014). Undiagnosed and untreated depression can reduce quality of life and encourage suicidal attempts in hemodialysis patients. Since sleep is essential to physical and psychological health in all individuals including hemodialysis patients (Čengić, Resić, Spasovski, Avdić, & Alajbegović, 2012), sleep disorders are regarded as a risk factor for the recurrence of major depression (Pigeon, 2010). Patients commonly complain about insomnia, restless leg syndrome, sleep apnea, and daytime drowsiness (Losso, Minhoto, & Riella, 2015). Although the etiology of sleep disorders in hemodialysis patients is unknown, their sleep quality is affected by physical factors (e.g. uremia, itching due to uremic pruritus (Pisoni et al., 2006), anemia, and metabolism of melatonin), psychological factors (e.g. anxiety, depression, and sleep disorders), and lifestyle-related factor (e.g. timing of hemodialysis and tea/coffee consumption) (Wang, Chan, Chang, Chen, & Tsai, 2013). The incidence of sleep disorders in these patients is accompanied by poor quality of life and high mortality rates (Elder et al., 2008; Turkmen et al., 2012). In spite of their high prevalence and importance, the vague symptoms of depression and sleep disorders prevent their timely diagnosis and treatment (Diefenthaeler, Wagner, Poli-de-Figueiredo, Zimmermann, & Saitovitch, 2008). The complex relationships of personal characteristics with poor sleep quality and depression, along with their effects on quality of life and mortality in hemodialysis patients, require extensive research. As very few studies have investigated the prevalence of poor sleep quality in hemodialysis patients, the relations between the mentioned condition and depression and other personal, clinical, and social conditions have not been fully addressed (especially in Iran). Thus, the present study aimed to evaluate the prevalence of depression and sleep quality disorders and their associations with some demographic and clinical features in hemodialysis patients.

## 2. Material and Methods

This descriptive correlational study was conducted on 310 patients undergoing hemodialysis in hemodialysis centers of eight educational hospitals in Mazandaran University of Medical Sciences (Sari, Iran) in 2014. The inclusion criteria were as follows: adult patients (age ≥ 18 years), who had received hemodialysis for at least three months were included if they were willing to participate, were aware of time and place, and underwent three-four-hours hemodialysis sessions two-three times in a week (all of them were outpatients). The exclusion criteria were as follows: Individuals who are travelers from other cities or received emergency dialysis during their hospitalization in other wards. The participants were ensured about the confidentiality of data and were explained about how to complete questionnaires. Next, data were collected using a demographic and clinical questionnaire, Beck Depression Inventory (BDI), and Pittsburgh Sleep Quality Index (PSQI). The demographic and clinical questionnaire contained questions about age, gender, marital status, employment, and education level, place of residence, dialysis frequency, HD duration, dialysis vintage, comorbidities, addiction, consumption habits, Body mass index (BMI), HD adequacy (Kt/V), and blood biochemical parameters. BDI contains 21 items, and uses 0-3 Likert scales, with total scores ranging from 0 to 63. In hemodialysis patients, scores above 15 indicate depression. Level of depression is assessed of BDI as follows: scores 0 to 15 means no depression, 16 to 32 means mild depression, 33 to 47 means moderate depression, and 48 to 63 means severe depression. BDI Intra-class Correlation Coefficient (ICC) has been found 0.85, and the validity of the scale was reported 0.81 using Spearman-Brown equation (Teles et al., 2014). In this study reliability of this questionnaire was confirmed with Cronbach’s alpha 0.92. Pittsburgh Sleep Quality Index (PSQI) includes 19 items, and assesses quality of patients’ nocturnal sleep during the past one month. This questionnaire includes 7 components of sleep, including subjective sleep quality, sleep latency, and sleep duration, habitual sleep efficiency, sleep disturbance, daytime dysfunction, and using sleeping medication. Total score of this index varies from 0 to 21 and PSQI≤5 mean adequate sleep, and PSQI>5 mean inadequate sleep quality. Instrument reliability was confirmed with Cronbach’s alpha 0.8 (Sabet, Naghizadeh, & Azari, 2012) and In this study, reliability was ensured with Cronbach’s alpha 0.72. Data were analyzed in SPSS-18 software. Descriptive statistics were used to analyze demographic and clinical data, Chi-Square test to find the relationship between qualitative variables, and regression model to assess the relationship of depression and sleep quality with other variables. P<0.05 was considered significant level.

## 3. Results

Of the 310 hemodialysis patients, 52.3% were men, with mean age 59.64 years. The majority of patients were married (78.1%), and illiterate (47.7%), and only 11.2% were employed. 80.3% of patients underwent hemodialysis three times per week, each time taking 3 to 4 hours in the daytime (96.1%). Mean dialysis vintage that patients underwent hemodialysis was found 39.89 months (Table 1).

**Table 1:** Sociodemographic and clinical data

Variables	N (%)	Means± SD	Variables	N (%)	Means± SD
**Age**(years)		59.64±13.94	**Dialysis Vintage**(months)		39.89±42.59
**Gender**			**Kt/V<1/2**	241(77.7)	
Male	162(52.3)				
female	148(47.7)				
**Marital status**			**Addict**	15(4.8)	
Single	20(6.5)		Smoke	13(3.1)	
Married	242(78.1)		Opium	1(0.3)	
Widowed	40(12.9)		Alcohol	68(21.9)	
divorced	8(2.6)		Medication	213(68.7)	
			No Addict		
**Educational Level**			**BMI**(Kg/m^2^)		
No Literacy	148(47.7)		Under weight	8(2.6)	
Literacy	88(28.4)		normal weight	138(44.5)	
High school	45(14.5)		over weight	103(33.2)	
College	29(9.4)		obesity	61 (19.7)	
**Employment status**			**PTH**(pg/ml)		
Employed	38(11.2)		Under 15	2(0.6)	
Unemployed	273(87.7)		15-65	32(10.3)	
			Above 65	276(89.1)	
**HD location**			**Hemoglobin**(g/dl)		
Town	177(57.1)		Low	288(92.2)	
village	133(42.6)		Normal	20(6.5)	
			High	2(0.6)	
**Dialysis frequency**	59(18.7)		**BUN**(mg/dl)		96.35± 42.11
2 a week	250(80.3)				
3 a week			**Creatinine**(mg/dl)		9.4±22.5
**HD duration**			**Phosphorus**(mg/dl)		5.79± 1.97
2 until 3 h a day	9(2.6)				
3 until 4 h a day	299(96.1)		**Calcium**(mg/dl)		11.71±51.08
**Consumption Habits**	239(77.1)		**Albumin**(g/dl)		4.24± 0.59
Tea drinking	4(6)				
coffee drinking	67(21.6)				
No habit					

In this study, 139 (44.8%) patients were found affected by depression. Among them 120 (38.7% of all the patients) had mild depression, 17 (5.5%) had moderate depression and 2 (0.6%) had severe depression. Among clinical factors, depression was only significantly related to level of BUN, phosphorus, and BMI>30. Depression decreased by 1.7% for every unit increase in BUN (P=0.001), and one unit increase in phosphorus will increase the depression up to 30% in (P=0.002). Obese patients with BMI>30 were suffering from depression (P=0.047) (Table 2).

**Table 2:** Predicators of depression examined by multiple regression

Variables	Unstandardized coefficients (B)	Standardized coefficients (Bate)	95% CI	p-Value
Age	0.012	0.012	-0.01152-0.03552	0.328
Gender	0.286	0.345	-0.3902-1.0212	0.40
Dialysis frequency	0.576	0.458	-0.32168-1.47368	0.20
BDI Score	0.376	0.364	-0.33744-1.08944	0.30
PSQI	0.027	0.379	-0.71584-0.76984	0.54
BUN	0.017	0.005	0.0072-0.0268	0.001
Phosphorus 0.087	0.267	0.087	0.09648-0.43752	0/002
BMI	0.694	0.349	1.010 - 3.967	0.047
Albumin	0.320	0.226	0.884 - 2.146	0.157
HB	0.265	1.466	0.043 - 13.582	0.857
Kt/V	0.509	0.305	0.331 -1.093	0.095

The results obtained showed 228 patients (73.5%) had poor sleep quality (average 9.2±4.4), and odds of poor sleep quality increased by 2% for every one year’s increase in age (P=0.048). Also, women were more likely to have poor sleep quality than men by 85% (P=0.04). In addition, poor sleep quality reduced by 2.5 times in those who underwent hemodialysis three times weekly (P=0.035). Sleep disturbance increased by 13% for every unit of increase in depression score (P>0.001) (Table 3).

**Table 3:** Predicators of sleep quality examined by multiple regressions

Variables	Unstandardized coefficients (B)	Standardized coefficients (Bate)	95% CI	p-Value
Age	0.021	0.011	-0.00056-0.04256	0.048
Gender	0.616	0.301	0.02604-1.20596	0.041
Dialysis frequency	0.878	0.416	-1.69336-0.06264	0.035
Dialysis Vintage	0.003	0.004	-0.01084-0.00484	0.436
Cr	0.014	0.014	-0.04144-0.04144	0.311
BDI Score	0.125	0.020	0.0858-0.1642	0.000

## 4. Discussion

Various studies have reported the prevalence of depression differently in hemodialysis patients. This might be attributed to the wide range of prevalence found using various mood disorder assessment tools (Chilcot, Wellsted, Da Silva-Gane, & Farrington, 2008). The present study results showed that 44.8% of patients undergoing hemodialysis suffered some degrees of depression, and 38.7% experienced mild depression, 5.5% moderate, and 6% severe depression. Numerous studies have found the prevalence of depression in patients undergoing hemodialysis from 43% (Simic et al., 2009) to 72% (Joshwa, Khakha, & Mahajan, 2012). In the majority of these studies, patients were younger than 65 years of age, or sample size had been small. In the present study, despite high prevalence of depression, no significant relationship was found between depression and demographic parameters. But there are significantly relationship between depression and clinical parameters such as: BMI>30, high blood phosphorus and reduced blood urea. Sanathan et al. argued that depression was related to less than normal BMI, and reduced survival, increased hospitalization, and increased mortality (Sanathan et al., 2014). It should be also noted that unlike dialysis population, increased BMI in the general population increases cardiovascular diseases, mortality rates and depression. In their study, Suzuki et al. (2011) cited that there is the significant relationship between restless leg syndrome (RLS) and depression with increased blood phosphorus (Suzuki et al., 2011). Teles et al. (2014), depression increased by decreasing phosphorus, they stated it was attributed to possible malnutrition and reduced albumin too (Teles et al., 2014). It seems, increased phosphorus in hemodialysis patients is due to non-compliance with low-phosphorus dietary regimen and not using phosphor-reducing medication following depression. Also depression in hemodialysis patients increased with reduced blood urea. It is seems depression symptom can be hidden with severe physical symptom (loss of appetite, sleeping problems etc.) due to psychological complication of uremia in the patients. Knuth et al. (2014) attributed the relationship of depression with reduced blood urea to lower food intake and increased catabolism of amino acids (Knuth et al., 2014). In the present study, no significant relationship was found between depression and albumin, KTV, or hemoglobin. Trbojevic et al. (2014) found depression related to age and reduced adequacy of dialysis, but there is not relationship with other demographic parameters, blood phosphorus or urea (Trbojević-Stanković et al., 2014). Micozkadioglu et al. (2006) and Dogan et al. (2005) found depression related to reduced hemoglobin and albumin (Araujo et al., 2012; Bornivelli, Aperis, Giannikouris, Paliouras, & Alivanis, 2012). In the present study population, the majority of patients appear to have been suffering from anemia and dialysis inadequacy. However, in the present study, lack of a significant relationship between depression and serum albumin may be due to better nutrition intake in participating hemodialysis patients and higher level of albumin compared to previous studies. In the present study, poor sleep quality increased with aging.

Cengic et al. (2010) stated that older people were more depressed than younger ones (Čengić & Resić, 2010). It seems older people are faced with more stressors, and suffer numerous chronic diseases such as diabetes mellitus, hypertension, and heart failure. In the present study, female patients had poorer quality of sleep. Sekercioglu et al. (2015) and Einollahi et al. (2015) reported that women had poorer quality of sleep (Einollahi, Motalebi, Rostami, Nemati, & Salesi, 2015; Sekercioglu, Curtis, Murphy, & Barrett, 2015). In the present study, patients who have a better sleep underwent hemodialysis three times per week. It seems Patients appear to enjoy better quality of sleep with increased frequency of hemodialysis per week and removal of toxins. In the present study, quality of sleep was not significantly related to the number of months of dialysis. Menon et al. (2015) reported poor quality of sleep in patients with more than three years history of hemodialysis (Menon et al., 2015).

## 5. Conclusion

Depression and poor sleep quality are two factors associated with hemodialysis patients, but patients do not overtly exhibit symptoms of these disorders. Considering the prevalence of depression in hemodialysis patients and its relationship with some laboratory test parameters, it is recommended that greater attention should be paid to regular measuring of these parameters, so that people at risk can be identified and treated. However, further studies are required to assess the relationship between these parameters and level of depression. Furthermore, because of the significant relationship between depression and quality of sleep, greater attention should be paid to these factors in elderly patients, women, and those undergoing shorter periods of hemodialysis per week.
